# Effects of stereopsis on vection, presence and cybersickness in head-mounted display (HMD) virtual reality

**DOI:** 10.1038/s41598-021-89751-x

**Published:** 2021-06-11

**Authors:** Wilson Luu, Barbara Zangerl, Michael Kalloniatis, Juno Kim

**Affiliations:** 1 School of Optometry and Vision Science, University of New South Wales (UNSW Sydney), Kensington, Australia; 2 Centre for Eye Health, University of New South Wales (UNSW Sydney), Kensington, Australia

**Keywords:** Sensory processing, Visual system, Cognitive neuroscience, Perception, Neuroscience, Displays

## Abstract

Stereopsis provides critical information for the spatial visual perception of object form and motion. We used virtual reality as a tool to understand the role of global stereopsis in the visual perception of self-motion and spatial presence using virtual environments experienced through head-mounted displays (HMDs). Participants viewed radially expanding optic flow simulating different speeds of self-motion in depth, which generated the illusion of self-motion in depth (i.e., linear vection). Displays were viewed with the head either stationary (passive radial flow) or laterally swaying to the beat of a metronome (active conditions). Multisensory conflict was imposed in active conditions by presenting displays that either: (i) compensated for head movement (active compensation condition), or (ii) presented pure radial flow with no compensation during head movement (active no compensation condition). In Experiment 1, impairing stereopsis by anisometropic suppression in healthy participants generated declines in reported vection strength, spatial presence and severity of cybersickness. In Experiment 2, vection and presence ratings were compared between participants with and without clinically-defined global stereopsis. Participants without global stereopsis generated impaired vection and presence similarly to those found in Experiment 1 by subjects with induced stereopsis impairment. We find that reducing global stereopsis can have benefits of reducing cybersickness, but has adverse effects on aspects of self-motion perception in HMD VR.

## Introduction

Visual motion perception is vital for interacting with the external world by providing informed feedback on self-motion. However, this is not routinely assessed clinically, where the focus is primarily on visual acuity and visual fields. Previous research has found a relationship between vision function changes (e.g., visual acuity, stereopsis and contrast sensitivity) and an increased likelihood of falls^1^ and reduced quality of life^2^. These changes may be caused by uncorrected refractive error, amblyopia/strabismus, cataracts and irreversible conditions such as age-related macular degeneration and glaucoma. For example, patients with cataracts reported greater perceived handicap levels and worse quality of life as a result of impaired stereopsis compared with declines in visual acuity^2^. Although these conditions may occur bilaterally, the severity may be greater in one eye resulting in differences in visual information perceived by each eye. This asymmetry in perception will lead to impaired stereopsis^3^.

Stereopsis is the ability to perceive depth and 3D structure binocularly and plays a role in spatial visual perception, including the perception of object-motion and self-motion^4–6^. One method to investigate the impact of stereopsis on motion perception is by applying anisometropic suppression. Anisometropic suppression occurs when one eye is defocused relative to the other, resulting in the visual field of one eye being partly or wholly suppressed. The prevalence of uncorrected anisometropia is not uncommon; Ostadimoghaddam and colleagues^7^ reported anisometropia of at least 1 dioptre in up to 7.3% of the population, which increases the risk of amblyopia development^8^. Pianta and Kalloniatis^3^ investigated behavioural effects of anisometropic suppression and found a negative impact on reaction time and threshold elevation. By inducing anisometropic suppression, which can be confidently achieved with at least 2 dioptres of anisometropia, stereopsis can be temporarily and artificially impaired. There is, however, limited literature on how stereopsis impacts visual motion perception when using head-mounted display (HMD) virtual reality (VR). HMD VR is a versatile investigative tool that allows for easy manipulation of motion stimuli in a safe and immersive environment and allows for the creation of a 3D rendered virtual space.

Compelling experiences of self-motion perception can be generated using HMD VR by simulating “optic flow”—the rich source of visual information about the *relative* motion of objects and self-motion^9^. Optic flow (see Supplementary S1) generates retinal motion, which is critical for the perception of ego-motion of moving organisms^9,10^. This retinal motion can be used to perceptually infer the motion of an observer generated relative to stationary or moving objects, surfaces, edges, light sources and any other visually salient contrasts. The retinal motion generated by optic flow can induce the experience of *vection*—the illusion of self-motion by completely stationary observers when they view optic flow displays^11^.

Studying vection has become important for understanding self-motion perception in both real and virtual environments. Any deficits in processing optic flow information should distort self-motion perception. Studies have found that vection strength is influenced by changes in the pattern of retinal motion^12–14^. It was found that reducing retinal adaptation to visual motion, such as by oscillating around a fixation point, appeared to help sustain the sensitivity to retinal motion stimulation caused by optic flow, which generated stronger overall vection^12–14^. Eye-movements also affect retinal motion and displacement of the focus of expansion, which in turn, can enhance the overall strength of vection^15–19^. These biases in the perceptual estimation of speed and direction of self-movements can also influence gait and walking speeds^20^. Hence, the visual perception of self-motion and behavioural responses to this information depends not just on optic flow, but also on the complex pattern of eye movements and retinal motion generated.

Although VR is a convenient and safe method for systematically altering retinal motion patterns generated by optic flow when observers are immersed in realistic virtual environments^21,22^, only recently have there been studies using HMDs to look at vection in depth^11,23,24^. Previous research focussed on presence, the experience of “being there” in the virtual environment^25^. Recently, Kim and colleagues^26^ found that the magnitude of spatial presence declined under conditions where cybersickness (adverse effects of nausea, dizziness, headache, disorientation, vomiting and other asthenopic and motion sickness-like symptoms) had reportedly increased. The concept of cybersickness is similar to motion sickness, however, it primarily depends on the presentation of visual motion in consistency with self-motion^23,27^. Previous studies showed that a sensory mismatch between physical and perceived head movements strongly contributed to symptoms of cybersickness^23,26,28,29^. Other vision-related factors appear to be involved in the generation of cybersickness when viewing dynamic virtual environments. For example, monocular viewing reduces the severity of cybersickness when display lag is increased^30^. This suggests that there may be a potential protective effect by having an absence of stereopsis. However, despite there being numerous studies investigating vection, spatial presence, and cybersickness in HMDs, there is limited research in this area involving participants who have global stereo-impairment.

The aim of the present study was to use virtual reality to understand the role of global stereopsis in the visual perception of self-motion. Our secondary aim was to further investigate the impact of head movements on visually perceived self-motion. We used HMD VR to present optic flow simulating self-motion in depth. We examined the effect of stereoscopic viewing on vection, presence and cybersickness measures in healthy individuals (see “Methods”). Anisometropic suppression was performed, as a non-invasive way to artificially impair stereopsis, to tease apart the contribution of global stereopsis to the experience of vection, presence and cybersickness in an immersive virtual environment (Experiment 1). We hypothesise that anisometropic suppression would reduce vection strength, presence and cybersickness compared with stereoscopic viewing. We also considered whether patients without clinically defined global stereopsis exhibited similar effects on vection and presence (Experiment 2). We hypothesised that if global stereopsis is important for vection, then we would further expect that patients without clinically defined global stereopsis will also have reduced vection and presence. To address our secondary aim, observers either viewed the displays with a stationary head posture (passive pure radial flow) or actively with predominantly linear inter-aural head movements. Active conditions simulated visual motion that were either compensated for ecological head displacement (active compensated) or did not compensate for head displacement (active pure radial flow). This was done systematically to vary the degree of visual-vestibular sensory conflict. We hypothesised that an increase in sensory conflict would reduce perceived vection and presence whilst increasing severity of cybersickness.

## Results

**Figure 1:**
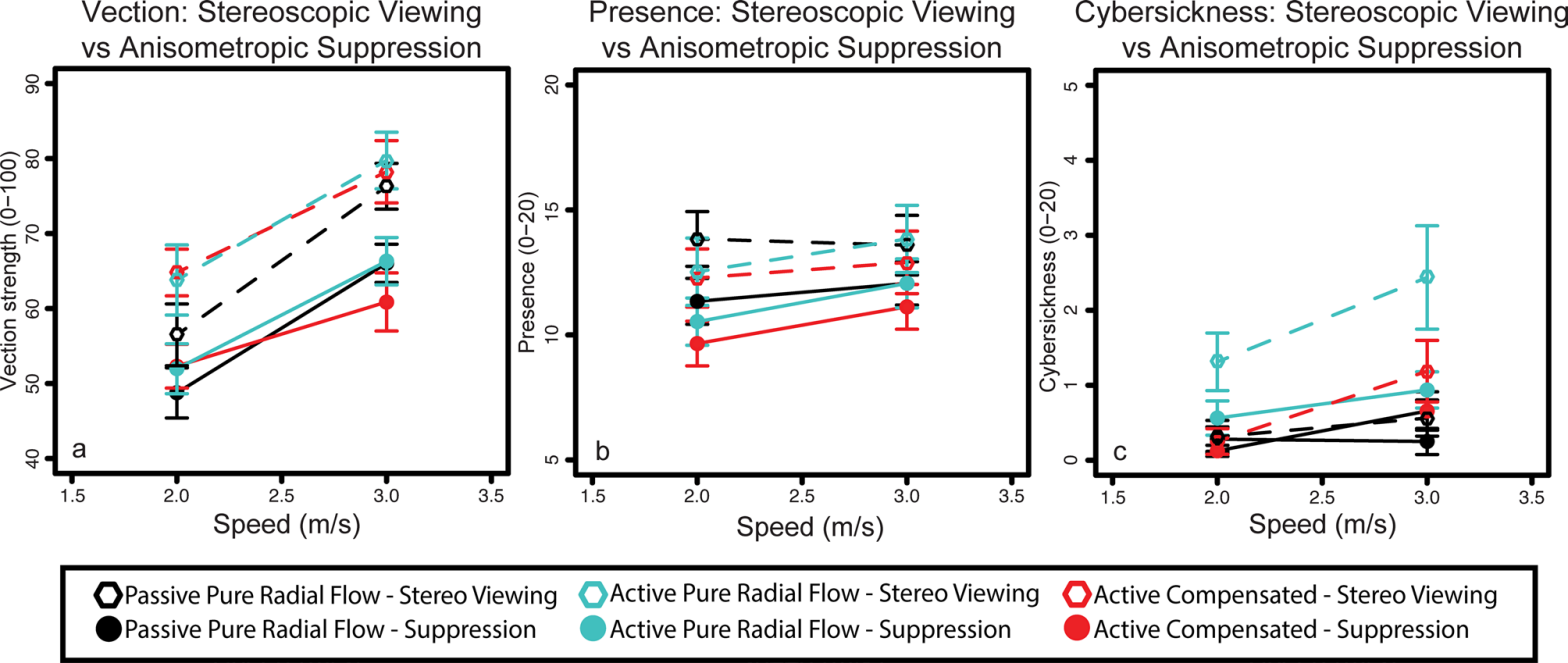
Mean vection strength (**a**), spatial presence (**b**) and cybersickness (**c**) scores plotted as function of simulated speed for each of the visual motion (passive radial, active radial and active compensation) and viewing conditions (stereoscopic viewing or anisometropic suppression). Open symbols represent results from stereoscopic viewing conditions and solid symbols represent results from viewing conditions following anisometropic suppression. Error bars show standard error of the mean. N = 32.

We first examined whether our dependent measures varied across the three active/passive viewing conditions and the two simulated speeds of Experiment 1. In Fig. 1, open symbols represent the group with stereoscopic viewing while filled symbols represent the group with anisometropic suppression. Three-way repeated-measures ANOVA were performed for each dependent measure. Perceived vection strength increased with increasing simulated speed (F_1,15_ = 36.36, p < 0.00005) and was higher during stereoscopic viewing compared with anisometropic suppression (F_1,15_ = 19.01, p < 0.005) (Fig. 1a). Perceived spatial presence slightly increased with increasing simulated speed (F_1,15_ = 5.215, p < 0.05) and during stereoscopic viewing when compared with anisometropic suppression (F_1,15_ = 9.582, p < 0.05) (Fig. 1b). Cybersickness increased with simulated speeds (F_1,15_ = 15.89, p < 0.005). Stereoscopic viewing induced greater levels of cybersickness compared with anisometropic suppression (F_1,15_ = 6.467, p =  < 0.05) (Fig. 1c). There was a two-way interaction between simulation speed and viewing condition on cybersickness (F_2,30_ = 5.334, p < 0.05) and between simulation speed and stereopsis on cybersickness (F_1,15_ = 5.116, p < 0.05). The active pure radial flow viewing condition appears to generate more cybersickness than other conditions (Fig. 1c). No other interactions were found.

In Experiment 2, we compared stereoscopic viewing of optic flow in participants with and without clinically defined global stereopsis to determine whether stereopsis affects perceived vection strength and spatial presence. The primary outcome focused on perceived spatial presence and perceived vection strength, while cybersickness was not evaluated in this experiment. In the previous experiment, we found main effects of simulated speed on spatial presence and vection strength. It is possible the effect might become more pertinent over a larger range of simulated speeds of self-motion in depth. Given the effect of anisometropic suppression in the previous experiment on reducing vection strength and spatial presence ratings, it is possible these perceptual declines will also be observed in patients who are clinically defined to have an absence of global stereopsis. We examined whether these two psychological attributes varied across the three active/passive viewing conditions and a wider range of four simulated speeds of self-motion (0–3 m/s).

**Figure 2:**
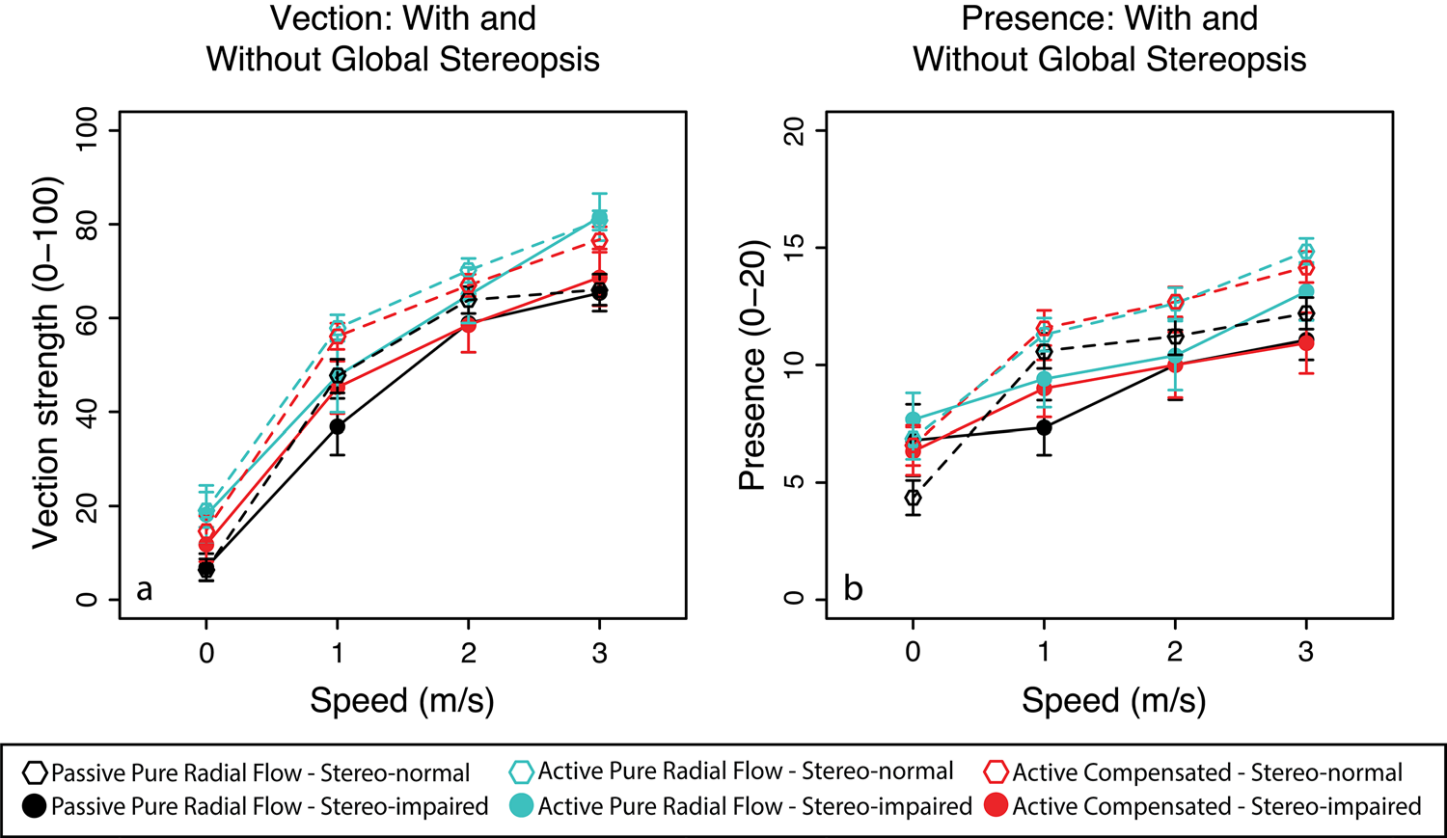
Mean vection strength (**a**) and spatial presence (**b**) scores plotted as function of simulated speed for each of the visual motion (passive radial, active radial and active compensation) and viewing conditions (with and without global stereopsis). Open symbols represent participants with global stereopsis while filled symbols represent those without global stereopsis. Error bars show standard error of the mean. N = 60.

Figure 2 shows the mean responses of perceived vection ratings (Fig. 2a) and perceived spatial presence (Fig. 2b) plotted against simulated speeds for each viewing condition (active pure radial flow, active compensated and passive pure radial flow). Main effects of simulation speed (F_3,129_ = 289.08, p < 0.00005) (F_3,129_ = 94.07, p < 0.00005) and viewing condition (F_2,86_ = 21.61, p < 0.00005) (F_2,86_ = 15.67, p < 0.00005) on perceived vection strength and spatial presence were found within subjects using a three-way mixed-effects ANOVA respectively. There were interactions between simulated speeds and viewing conditions (F_6,258_ = 2.15, p = < 0.05). There were also interactions between global stereopsis and simulated speeds on perceived spatial presence (F_3,129_ = 3.48, p < 0.05). These results show there are significant differences in vection strength and spatial presence for both changes in simulated speed of self-motion and viewing condition.

**Figure 3:**
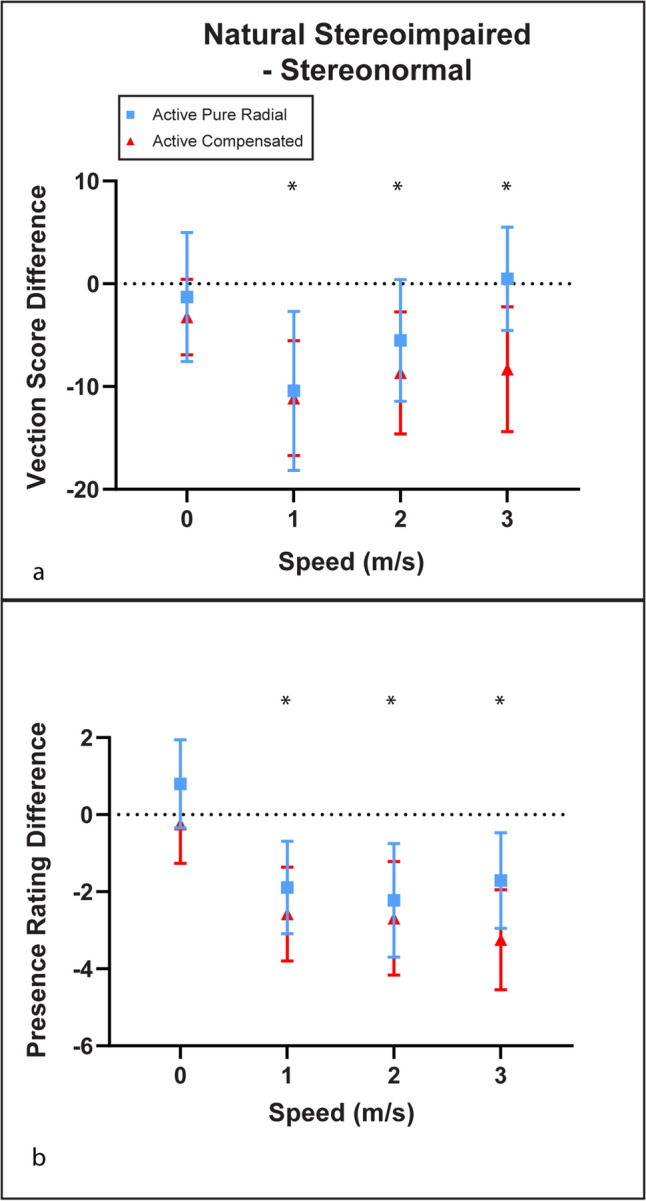
Mean difference plots for vection score (**a**) and spatial presence rating (**b**) comparing those without global stereopsis with stereo-normal during active display configurations in Experiment 2. Ordinates represent differences compared to normal stereopsis. Positive differences indicate advantage over normal stereopsis. Negative differences indicate disadvantage compared with normal stereopsis. Error bars represent 95% confidence interval. ‘*’ represent p < 0.05.

We performed further analyses to compare the differences of vection strength scores and perceived spatial presence in active conditions in those with normal and without global stereopsis (see Fig. 3). Those without global stereopsis reported relatively lower vection scores in active compensated conditions compared with those with global stereopsis (M = − 8.31, 95% CI [− 14.25, − 2.37]) (Fig. 3a). This also occurred during the active pure radial flow condition in the slowest simulated speed (M = − 10.42, 95% CI [− 18.00, − 2.84]), however, the differences become less apparent as the speed increased. Those without global stereopsis also reported lower perceived spatial presence regardless of display compensation in all moving speeds (M = − 3.24, 95% CI [− 4.51, − 1.97]) (Fig. 3b). No differences were found in both vection scores and perceived spatial presence when there was no simulated speed.

The results show a significant decline in vection strength and spatial presence when imposing simulated self-motion, compared with stationary simulations. These declines in vection strength were most consistent for active compensated displays and were not significant for the higher simulated speeds with active viewing of pure radial flow. The declines in spatial presence during simulation of self-motion was consistent across both active viewing conditions.

**Figure 4:**
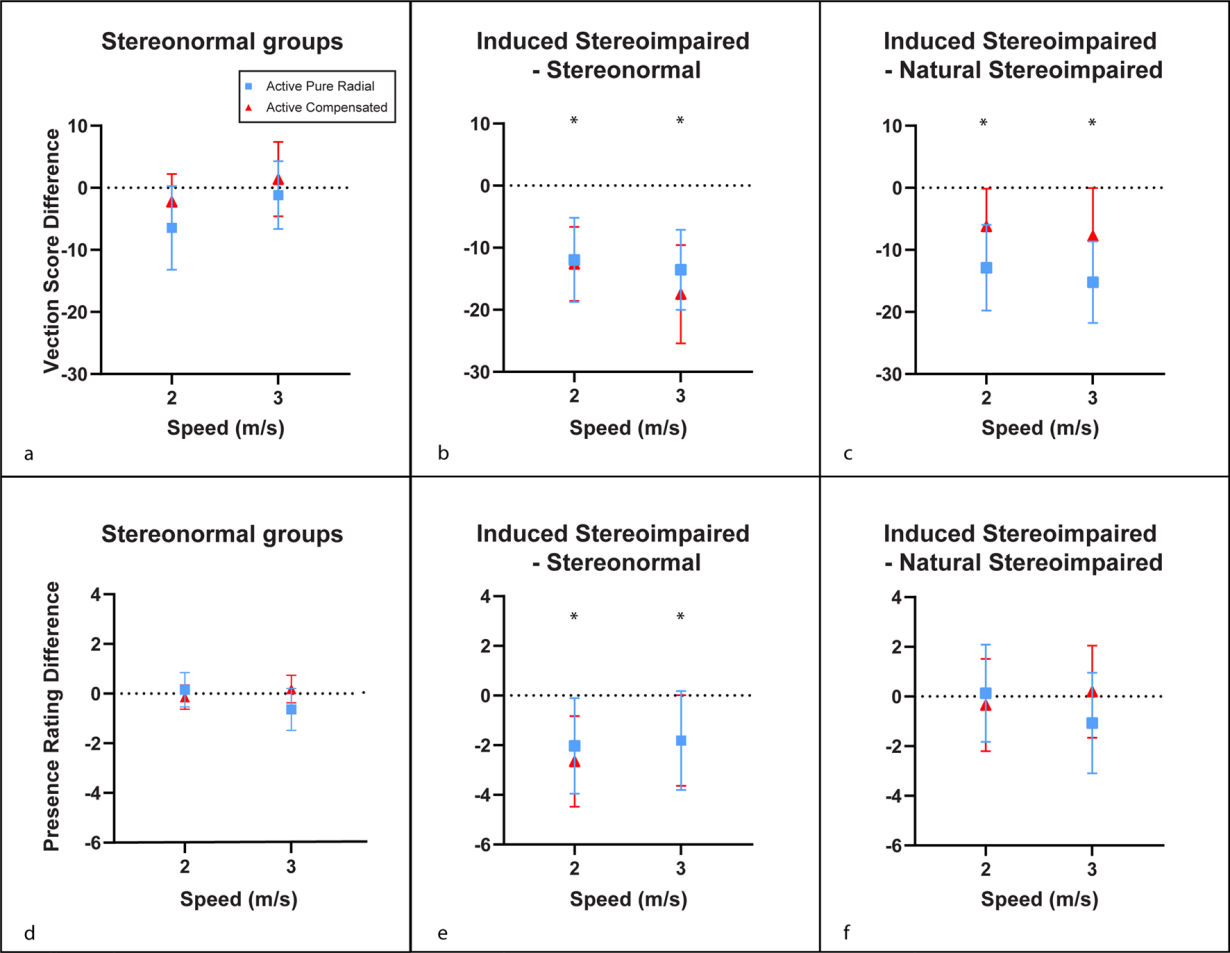
Mean difference plots for vection score (top row) and spatial presence rating (bottom row) during active display configurations. (**a**) and (**d**) compare data on the stereo-normal group before anisometropic suppression in Experiment 1 and the stereo-normal group in Experiment 2; (**b**) and (**e**) show the within-subjects comparison of participants with and without anisometropic suppression in Experiment 1; (**c**) and (**f**) show differences obtained with induced anisometropic suppression relative to participants who naturally do not have global stereopsis. Positive differences indicate advantage of the first listed group compared with the second listed group. Negative differences indicate disadvantage of the first listed group compared with the second listed group. Error bars represent 95% confidence interval. ‘*’ represent p < 0.05.

We compared the stereo-normal groups and those without global stereopsis and those with induced stereo-impairment using difference plots in the active conditions. No difference in vection scores and spatial presence ratings were observed between stereo-normal groups as highlighted in Fig. 4a, d respectively. As previously discussed, those with induced anisometropic suppression reported lower vection scores as highlighted in Fig. 4b (M = − 11.97, 95% CI [− 18.49, − 5.45]). This effect was observed for spatial presence in both speeds during the active compensated condition (M = − 1.81, 95% CI [− 3.76, − 0.05]) and during the slower speed in the active radial condition (M = − 2.03, 95% CI [− 3.88, − 0.18]) (Fig. 4e). Those having been induced with anisometropic suppression reported lower vection scores compared with those without global stereopsis in both conditions (M = − 6.62, 95% CI [− 12.04, − 0.40]) (Fig. 4c). There was no difference between natural stereo-impairment and induced stereo-impairment in perceived spatial presence (Fig. 4f).

## Discussion

Our results show that impaired binocular vision through anisometropic suppression reduces perceived vection strength and spatial presence (Experiment 1). We also found strong interactions between global stereopsis and simulated speed of self-motion on vection strength, spatial presence and cybersickness. The imposed suppression of stereopsis was only temporary and did not allow enough time for habituation. We considered this suppression might be compensated for in individuals lacking global stereopsis for a long period of time. To this end, we recruited participants who were clinically defined by an absence of global stereopsis (Experiment 2) to ascertain the impact of established versus the ‘temporary’ stereo-suppression investigated in Experiment 1.

Experiment 2 found those without global stereopsis reported higher levels of experienced vection in the active viewing conditions compared to the anisometropic suppression group. A possible explanation may be that participants with longstanding absence of global stereopsis have habitually re-sensitised to visual motion processing. The effects on motion perception following acute stereo-suppression compared with long-term stereo-impairment highlights the potential impact of eye diseases on motion perception following rapid unilateral vision impairment (e.g., in age-related macular degeneration). However, coarse stereopsis may still be retained if there are corresponding points of intact retina in both eyes^31^. Further research is required to better understand disease processes on motion perception and is beyond this study.

To explain why perceived vection strength and spatial presence were lower during anisometropic suppression and those without global stereopsis, we propose that binocular vision provides several benefits in inferring scene layout. The lateral displacement of the eyes provides two disparate retinal images of the same object leading to benefits such as: increased field of view, binocular summation, stereopsis, improved information about environmental layout, depth and parallax, and improved perception of rigidity and structure^5^. Our findings are consistent with previous research which found that introducing consistent stereoscopic depth cues enhanced linear vection along different geographic trajectories^5,32–34^. However, these studies only examined the role of binocular vision and stereopsis on vection generated by frontal flat panel projection displays viewed through polarised glasses. These external displays subtended visual angles of up to 66° when viewed frontally from a distance. Keshavarz and colleagues^35^ found that viewing stimuli within large dome-like displays or multiple displays arranged in a three-dimensional (3D) structure resulted in stronger experiences of vection compared with a single central display. A recent study also highlighted the role of the peripheral visual field in the experience of a three-dimensional viewing environment, which enhanced vection strength and spatial presence perception^36^. Our HMD VR is able to present visual stimuli to up to 110° in angular viewing range, a presentation that encompasses a wider field of visual stimulation compared to frontally viewed flat panel displays. Alternatively, the disadvantages observed in perceived vection strength and spatial presence in Experiment 1 may be explained by the reduction in visual clarity in the “fogged” eye during induced anisometropic suppression, rather than impaired binocularity. This is a factor that should be investigated in future studies.

In investigating the effects of head movements, we found that active viewing conditions using an HMD enhanced vection strength compared with passive viewing in those with global stereopsis. This dependence of vection on visual-vestibular coupling differs from the findings of Kim and Palmisano^37^, in which no differences in vection were found between active and passive viewing conditions presented on an external display without fixation. The present study utilised a stationary fixation point to suppress ocular following responses (eye movements that are generated by large-field visual stimuli during self-motion to stabilise retinal images)^38^ and thus increases retinal motion. Rather than depending on retinal motion per se, we instead propose that vection is enhanced in active viewing conditions by the generation of motion parallax information that provides depth cues within the visual scene. This proposal is consistent with the results of a previous vection study using external displays^17,39^.

Building on previous vection studies^40,41^, it is possible that spatial presence enhances vection strength and vice versa. Spatial presence was enhanced by active viewing compared to passive viewing in those with global stereopsis. This increase in spatial presence was not observed in those without global stereopsis. Therefore, it appears that spatial presence is improved by stereoscopic depth cues and supports previous research by Ijsselsteijn and colleagues^42^. Surprisingly, they did not find a significant effect of stereoscopic viewing on vection strength despite increased perceived spatial presence. This difference may be attributed to the visual stimuli used, where their participants viewed a real-world stimulus projected on a large flat-panel display whereas we used an HMD with moving dots. As previously discussed, there are advantages of using an HMD over flat-panel displays^35,36^. Additionally, our HMD display was generated to surround and immerse the observer in VR to mimic realism within a three-dimensional space. Contrarily, Ling and colleagues^43^ found that stereo-ability and stereoacuity did not have an impact on spatial presence in a public speaking activity. A possible explanation may be that the simulated audience were largely physically distant from the observer (6.6–6.8 m away) resulting in limited differences in stereoscopic and non-stereoscopic rendering.

We found that global stereopsis increased cybersickness, supporting a previous report which found that monocular viewing reduces cybersickness severity^30^. Faster simulated speeds of self-motion also generated more symptoms of cybersickness and higher vection scores. These findings differed to Palmisano and colleagues^44^ who found no significant relationship between vection and cybersickness. This discrepancy may again be explained by differences in displays used, where they used screen-projected visual stimuli as opposed to HMD VR. Displays rendered in HMD VR allow for the integration of positional feedback from head movements which may limit sensory mismatch. The wider angle of visual stimulation in HMD VR compared with screen-projected visual stimuli could lead to stronger vection perception as aforementioned, which could result in a more significant relationship between vection and cybersickness. Cybersickness is also more likely to be experienced following active head movements, which is commonly attributed to display lag^26,29^. However, Feng and colleagues^28^ compared the Oculus Rift CV1 (our model) with the latest model Oculus Rift S and found that both devices were capable of achieving extremely low baseline lag (< 5 ms). This is lower than that of older systems that are known to generate strong compelling cybersickness like the Oculus Rift DK1^45^, which has a comparatively higher display lag (> 30 ms).

In conclusion, HMDs offer great utility to better understand motion processing in virtual environments for those with and without global stereopsis. This study highlights the effects of impaired stereopsis due to induced anisometropia on vection strength and spatial presence (and also cybersickness). This is the first study that we are aware of that investigates the effects of clinically-defined absence of global stereopsis on self-motion perception in virtual environments. Our findings indicate that by identifying and correcting anisometropia, the disadvantages of impaired stereopsis on vection strength and spatial presence can potentially be mitigated. However, this may have undesirable consequences of increased susceptibility to cybersickness whilst using HMD virtual reality. It would be of benefit in future to ascertain the extent to which other types of ocular disorders disrupt the sensation of retinal motion and bias perception of self-motion in realistic self-motion contexts.

## Method

### Participants

Seventy-six (76) naïve adults (37 females and 39 males) between the ages of 18 and 27 (19.54 ± 2.27) participated in this study. All observers were screened for their visual acuity using a standard Bailey-Lovie LogMAR Chart calibrated for 3 m, their stereo-acuity using a Random Dot Stereo Acuity Chart with Lea Symbols (Vision Assessment Corporation, 2007), observers’ pupillary distance and ocular health by an experienced clinician. All participants had visual acuities of LogMAR 0.3 (Snellen 6/12) or better in the worse eye with or without correction (legal requirement for private unconditional driving in Australia). All observers had no prior vestibular dysfunction and were not prone to motion sickness. Participants were excluded if they had a history of amblyopia or strabismus.

Sixty-one (61) participants could perceive global stereopsis. Of these, sixteen (16) participants observed the display under anisometropic suppression. Anisometropic suppression was confirmed with a follow-up stereo-acuity assessment. The remaining fifteen (15) could not perceive global stereopsis using the random dot stereogram. Participant characteristics are shown below in Table 1. Procedures were approved by the Human Research Ethics Advisory (HREA) panel at University of New South Wales (UNSW Sydney). Informed written consent was received by all participants and procedures were conducted in accordance with the HREA panel at UNSW Sydney guidelines and regulations and approved protocol.

**Table 1:** Characteristics of observers.

	Normal	Normal (before anisometropic suppression)	Stereo-impaired	Total
Participants	61	16	15	76
Mean age ± SD	19.33 ± 1.94	21.00 ± 1.46	20.40 ± 3.14	19.54 ± 2.27
No. of females (males)	31 (30)	8 (8)	6 (9)	37 (39)
Mean binocular visual acuity (LogMAR) ± SD	0 ± 0.04	0.00 ± 0.00	0.02 ± 0.04	0 ± 0.06
Inter-eye difference visual acuity (LogMAR) ± SD	0.03 ± 0.06	0.00 ± 0.00	0.09 ± 0.07	0.04 ± 0.07
Stereoacuity (Arcsec) ± SD	^†^52.67 ± 38.30	^†^36.73 ± 19.47	233.33 ± 121.47	^†^52.67 ± 38.30
Stereoacuity (Anisometropic Suppression) (Arcsec) ± SD	–	252.50 ± 100.72	–	–

^†^Mean stereoacuity and standard deviation calculated in participants who could perceive global stereopsis.

*SD* standard deviation.

### Display generation

Displays simulating illusory self-motion in depth (vection) whilst facing forward (pure radial optic flow) were created using our custom software developed using Visual C++ and Microsoft Visual Studio 2010. Displays were generated similarly to our recent works^36^. This software utilised OpenGL and the Oculus Rift CV1 SDK. A spherical 3D cloud (of radius approximately 3 m) was populated with 18,432 blue squares (ranging in optical size from 0.25° to 2.5° with proximity to the observer) and was simulated to surround the observer. The blue squares moved in a radial pattern from a focus of expansion towards the observer at varying stepwise speeds (ranging from 0 to 3 m/s). The observer perceives the motion of moving forward through the 3D cloud. A small green central target was used to orient observers to ensure they are facing the appropriate direction. A fixation target (small white dot) was set slightly below the focus of expansion. A baseline modulus was developed as a reference (passive viewing with set speed “2 m/s”) for observers to view prior to the trials. Each speed setting was calibrated so that the blue squares would travel in their respective metres/second (m/s). Three questions appear after each trial following each presentation to rate vection strength, spatial presence and cybersickness. Vection strength was rated on a vertical scale ranging from 0 (completely stationary) to 100 (if they felt they were moving like if they were sprinting). Spatial presence was rated on a vertical scale between 0 (completely disconnected from the virtual world and still feel like they are in the physical world) and 20 (completely within the virtual world). Cybersickness was rated based on the Fast Motion Sickness (FMS) scale, ranging from 0 (asymptomatic) to 20 (frank sickness resulting in emesis)^46^.

Experiment 1 was conducted with a total of 6 trials presented in randomised order. The faster two speeds (speed “2” and “3”) were used. Displays either generated pure radial optic flow without head movements (passive viewing), pure radial optic flow despite head movements (active uncompensated viewing) or were compensated for head movements consistent with a constant spatial direction of self-motion (active compensated viewing). The displays were rendered to incorporate observer head movement information in real time during compensated viewing conditions. An audible tone was delivered to the Rift’s earpiece at a rate of 1 Hz for four of the trials. The audible tone signalled for the observer to sway (active viewing) at the rate of metronomic sound. Yaw, pitch, and roll changes in head orientation were recorded for all trials using the Rift’s inherent accelerometers and gyros and were computed as Euler angles in degrees. Experiment 2 consists of a total of 12 trials (with all speed settings) presented in randomised order.

### Procedure

Two pairs of spectacles were made to provide a 3 dioptre difference between the two eyes. Each spectacle had opposite eyes ‘fogged’ to prevent any influence of accommodation. This was designed to induce anisometropic suppression and hence impair stereopsis^3^. Participants were asked to wear their contact lenses (if required correction) to ensure the 3 dioptre difference was retained. Prior to the trials, observers had their stereo-acuity measured with and without the spectacles to demonstrate the effect on global stereopsis. The spectacles were assigned randomly to participants to mitigate eye order effects.

The Oculus Rift (CV1) pupillary distance was adjusted based off the measurement found during screening. Each participant was seated and the HMD was placed on the observer until a comfortable fit was achieved. The observer was asked to adjust the head-mounted device vertically until the Oculus Home page was most clear. The device was then tightened to a comfortable point using the attached Velcro.

The experimenter instructed the observer of the steps involved in the example and experiment. The experimenter initiated the example modulus trial to the observer prior to the experimental trial to ensure they understood the task as well as provide a baseline comparison. In the first experiment, the HMD was then removed and the participants were asked to wear the first pair of anisometropic-suppressing spectacles. During each of the displays, the observer was asked to orient themselves using the green central target. They were then asked to fixate on the white fixation target during the presentation and concentrate on the experience (if any) of illusory self-motion in depth. The observer was asked to sway when an audible metronomic tone was heard in the earpiece of the Oculus Rift and remain stationary when there were no audible tones. The observer was asked to grade the level of vection strength, spatial presence and whether they felt sick in order to get used to method of answering using the directional keys and spacebar on the provided keyboard. After the first set, the observer was asked to remove both the HMD and the spectacles and the trials were repeated. This was then repeated again with the second pair of spectacles. In Experiment 2, participants only wore their optical correction (if any) and no anisometropic suppressing spectacles were used.

### Statistical analysis

Head movement amplitudes and frequencies were first analysed to ensure the effects on these measures were due to the presence or absence of stereopsis as oppose to eye order for anisometropic suppression or head movements (see Supplementary Information S2). We then compared stereoscopic viewing of optic flow in healthy participants with and without anisometropic suppression to determine whether stereopsis affects perceived vection strength, spatial presence and cybersickness (Experiment 1). In Experiment 2 we compared perceived vection strength and spatial presence in healthy participants and those without global stereopsis. Analyses were performed using the statistical programming language R (R version 3.6.1; Foundation for Statistical Computing, Vienna, Austria) and Graphpad Prism (Version 8.0.0; GraphPad Software, Inc., San Diego, U.S.A.).

## Supplementary Information

Supplementary Information.
