# Supplementary material for: Effects of stereopsis on vection, presence and cybersickness in head-mounted display (HMD) virtual reality
Source: Sci Rep. 2021 Jun 11;11:12373. doi: 10.1038/s41598-021-89751-x (PMC8196155; doi:10.1038/s41598-021-89751-x)
Supplement: Supplementary file 1 — Supplementary Information. [file 41598_2021_89751_MOESM1_ESM.pdf]

Supplementary Information

Manuscript: Effects of stereopsis on vection, presence and cybersickness in head-mounted display (HMD) virtual reality.

Authors: Wilson Luu[1,2], BOptom BSc, Barbara Zangerl[1,2], PhD, DVM, Michael Kalloniatis[1,2], PhD, *Juno Kim[1], PhD

1. School of Optometry and Vision Science, University of New South Wales (UNSW Sydney), Kensington, Australia

2. Centre for Eye Health, University of New South Wales (UNSW Sydney), Kensington, Australia

S1. Optic Flow

Optic flow information can be classified into several components, namely radial, circular (angular), translational (planar, linear or laminar) and sheer motion.[1-3] When an observer moves in a straight line the focus of expansion (FoE) from the direction of heading generates motion vectors which radially expand from the FoE (see Supplementary Fig. 1a). When the observer moves within the scene and changes the direction of gaze, an additional component of flow information is added which shifts the FoE relative to fixation (see Supplementary Fig. 1b). This shift in the FoE is due to a disruption of the retinal image.[4]

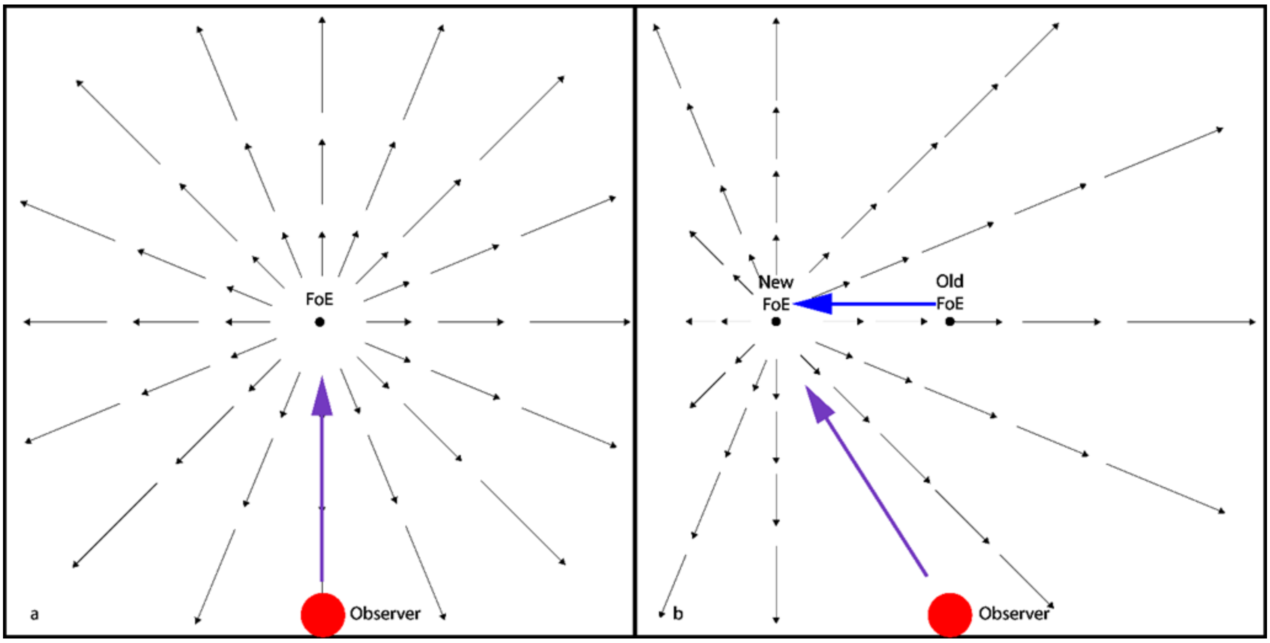

Supplementary Figure S1. Optic flow fields demonstrating FoE and influence of change in direction of gaze (heading) as observer moves forward. Red spot represents the observer. Black dot shows FoE. Black arrows represent direction of optic flow. Purple arrows represent direction of heading which is pointing towards the FoE. 1a) Pure radial optic flow pattern from the focus of expansion as observer moves forward. 1b) Optic flow fields have additional translational component due to change in direction of gaze. Blue arrow represents shift of the focus of expansion relative to direction of heading.

Supplementary Data

S2. Head-movement analysis

Head movement amplitudes and frequencies were analysed to ensure the effects on these measures were due to the presence or absence of stereopsis as opposed to eye order for anisometropic suppression or head movements. There were no significant differences between eye order for anisometropic suppression ($t_{15} = 0.90$, $p = 0.38$) or head movements ($t_{15} = 0.1$, $p = 0.92$) and hence scores following anisometropic suppression were averaged between both eyes. We examined the variability of head movements using three-way repeated-measures ANOVA and found a main effect of viewing condition on the amplitude of head oscillation ($F_{2,30} = 31.53$, $p < 0.00001$). No significant effects of anisometropic suppression and simulation speeds were found on head movements.

In Experiment 2, we also considered whether the amplitudes of inter-aural linear head movement were similar between the stereo-normal and stereo-impaired participants and found no between-subjects effect ($F_{1,58} = 0.05$, $p = 0.83$) using a three way mixed ANOVA. We also examined whether these head movements varied across the three viewing conditions and four speeds of simulated self-motion in depth and found a within-subjects main effect of viewing condition on the amplitude of head oscillation ($F_{2,116} = 127.42$, $p < 0.0001$). However, follow-up t-tests found no significant differences between the active compensation and no compensation conditions on the amplitude of head translation for the stereo-impaired participants (t_{14}=1.10, $p = 0.29$) and for the stereo-normal participants (t_{44}=0.98, $p = 0.41$) (Supplementary Fig S1). There were no significant effects of simulation speed on head movements. These results are consistent with the findings of Experiment 1.

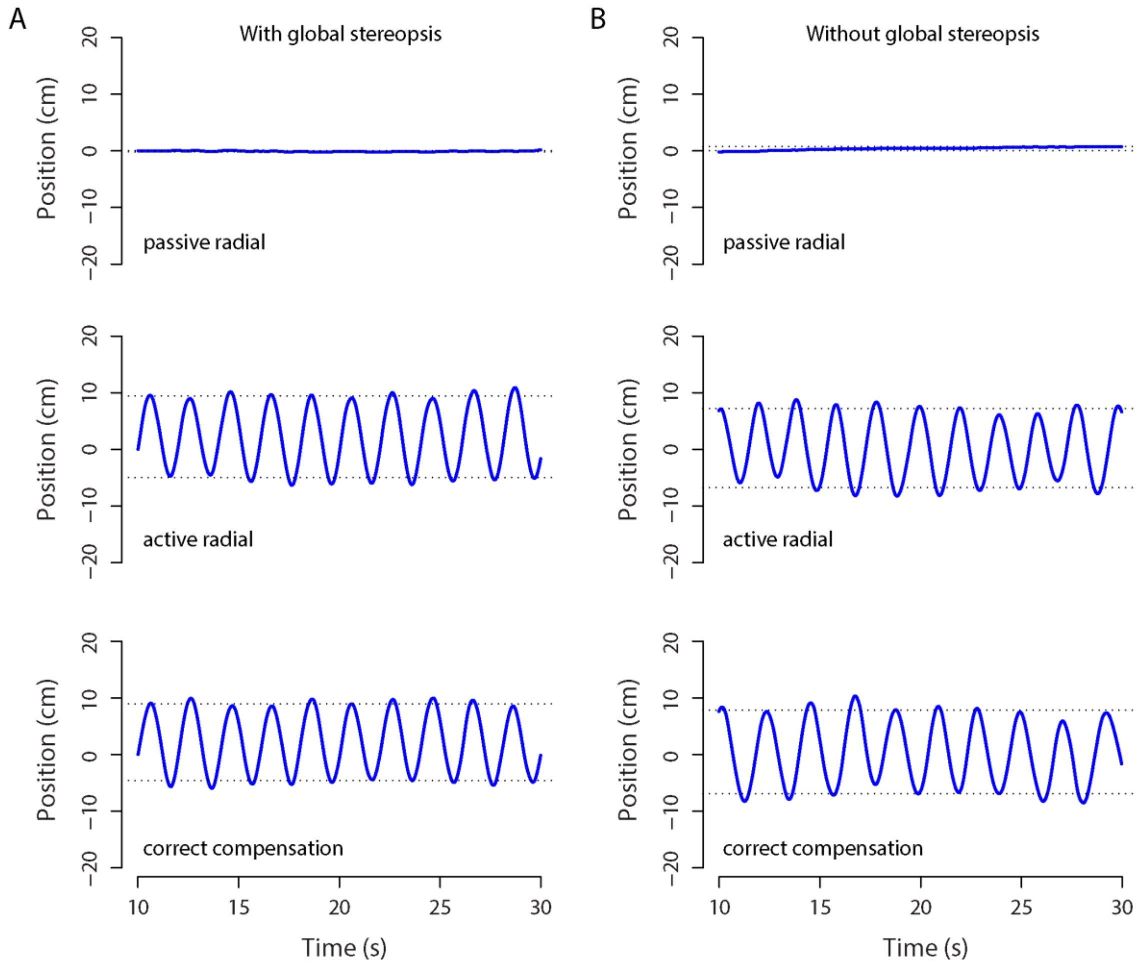

Supplementary Figure S2. Time-series plots of inter-aural head position for each of the three head movement conditions. Sample plots shown for two individuals with (left) and without (right) global stereopsis. Separate axes show data for the fast speed condition with passive viewing (upper), without compensation (middle) and with correct compensation (lower). Dashed lines show the results of the method used to estimate the overall range of head translation (±1.33 SDs).

References

1 Koenderink, J. J. Optic flow. *Vision Res* **26**, 161-179 (1986).
2 Morrone, M. C. *et al.* A cortical area that responds specifically to optic flow, revealed by fMRI. *Nat Neurosci* **3**, 1322-1328, doi:10.1038/81860 (2000).
3 Koenderink, J. J. & van Doorn, A. J. Facts on optic flow. *Biol Cybern* **56**, 247-254 (1987).
4 Bradley, D. C., Maxwell, M., Andersen, R. A., Banks, M. S. & Shenoy, K. V. Mechanisms of heading perception in primate visual cortex. *Science* **273**, 1544-1547 (1996).
